# Expression and localisation of osteopontin and prominin-1 (CD133) in patients with endometriosis

**DOI:** 10.3892/ijmm.2013.1325

**Published:** 2013-03-28

**Authors:** FABIO D’AMICO, EVANGELIA SKARMOUTSOU, GIUSEPPE QUADERNO, GRAZIA MALAPONTE, CARMELO LA CORTE, GIUSEPPE SCIBILIA, GABRIELLA D’AGATE, PAOLO SCOLLO, FILIPPO FRAGGETTA, DEMETRIOS A. SPANDIDOS, MARIA CLORINDA MAZZARINO

**Affiliations:** 1 Department of Biomedical Sciences, Pathology and Oncology Unit, University of Catania, Catania, Italy; 2 Pathology Unit, Cannizzaro Hospital, Catania, Italy; 3 Department of Obstetrics and Gynecology, Cannizzaro Hospital, Catania, Italy; 4 Laboratory of Clinical Virology, Faculty of Medicine, University of Crete, Heraklion, Crete, Greece

**Keywords:** osteopontin, CD133, endometrium, endometriosis, quantitative real-time RT-PCR, immunohistochemistry

## Abstract

In this study, we investigated the expression and localisation of the proteins, osteopontin (OPN) and prominin-1 (CD133), as well as the plasma OPN levels in the endometrium of patients with endometriosis. Samples of ectopic endometriotic lesions and normal endometrium were obtained from 31 women with endometriosis and 28 healthy control subjects. The mRNA and protein expression of OPN and CD133 was analysed by real-time RT-PCR and immunohistochemistry. The plasma levels of OPN were determined by ELISA. Our results revealed that OPN mRNA and protein expression, as well as its release in the blood, was significantly increased in the endometriotic lesions in comparison to normal tissue. Although the presence of CD133^+^ cells was detected in the normal endometrium, as well as in the endometriosis specimens, a significant quantitative variation of this protein was not demonstrated in the patients with endometriosis. In conclusion, our data indicate that OPN is involved in the development of endometriosis by enhancing the invasiveness, proliferation and survival of endometrial cells in ectopic lesions. CD133 cannot be used as a disease marker for endometriosis, although an involvement of this protein in the pathogenesis of endometriosis cannot be excluded.

## Introduction

Endometriosis is a complex and chronic gynecological disorder characterised by the presence of endometrial tissue outside the uterus (1). Genetic, hormonal and environmental factors contribute to the susceptibility to endometriosis; however, the pathogenesis of this disease has not yet been fully elucidated.

Although endometriotic cells are not characterised by uncontrolled proliferation, they show some properties of malignant tissues, such as invasion, induction of metastasis, and the ability to evade apoptosis (2,3). In particular, it is known that the ability of endometriotic cells to invade surrounding tissue is induced by a group of proteins termed metastasis-inducing proteins (MIPs), such as osteopontin (OPN) (4).

OPN, a 70-kDa secreted glycoprotein, is mainly involved in cell adhesion and migration (5), and it has been found to be expressed in endometrial epithelium in normally cycling fertile women (6). However, various studies on the endometrial expression of OPN in patients with endometriosis have provided controversial results. A previous study demonstrated that the OPN protein is densely expressed in eutopic normal endometrium, as well as in epithelial cells of endometriotic cysts (7). Moreover, OPN mRNA expression, as well as its plasma levels, have been shown to be higher in patients with endometriosis compared to normal subjects (8). It has been reported that OPN mRNA levels are reduced during the early secretory phase of women with moderate-to-severe endometriosis (9,10).

Another feature of endometriosis is represented by its stem cell origin (11). It has been hypothesised that endometriosis may be caused by a dysregulation of stem cell function (12).

Prominin-1 (CD133), a stem cell-associated antigen, is a 120-kDa glycoprotein, and a member of the prominin family of pentaspan membrane proteins (13). CD133 has been shown to be localised in glandular and luminal epithelial cells of the normal endometrium (14).

The spreading of endometrial epithelial progenitor cells may represent one of the mechanisms involved in the pathogenesis of endometriosis, a disease characterised by a dense vascularisation of its lesions (15). It is known that OPN may influence the angiogenesis, proliferation and migration of endothelial progenitor cells, acting as a regulator of CD133^+^ progenitor cells (16,17).

The present study aimed to determine whether OPN and CD133 expression is altered in the human ectopic endometrium, and whether the expression of these two molecules correlates with the clinical features of endometriosis. The expression profiles of OPN and CD133 were analysed in ectopic lesions, as well as in normal endometrium by real-time RT-PCR and immunohistochemistry. Furthermore, we also evaluated the plasma levels of OPN in patients with endometriosis.

## Materials and methods

### Patient selection

Sixty-one women were enrolled in this study after providing written informed consent. Thirty-one patients underwent laparoscopic surgery at the Department of Obstetrics and Gynecology, Cannizzaro Hospital, Catania, Italy. As control subjects, 30 women with benign non-endometriotic ovarian cysts were enrolled in this study. Clinical data including, age, history of pregnancy, parity, body mass index (BMI) and serum CA125 levels were collected at surgery. Endometriosis was confirmed by a histopathological examination of samples and the extent of the disease was evaluated according to the revised classification of endometriosis provided by the American Society of Reproductive Medicine (18). Twenty-two cases were classified as minimal-to-mild disease (stage I and II) and 9 cases were classified as moderate-to-severe disease (stage III and IV). All the patients were in the proliferative phase of the menstrual cycle. The study protocol was approved by the local ethics committee.

### RNA extraction and real-time RT-PCR

Fresh endometrial specimens were immediately transferred in RNAlater™ (Sigma-Aldrich, St. Louis, MO, USA) and stored at −80°C until RNA extraction. Tissue specimens were pulverised and then dissolved in TRIzol reagent (Invitrogen, Carlsbad, CA, USA), according the manufacturer’s instructions. The concentration of the purified RNA was determined by spectrophotometry. For further analysis, equal RNA loading and integrity was confirmed by showing consistent intensities of 28S and 18S rRNA bands on RNase-free agarose gel electrophoresis. A total of 2 μg of RNA from each sample was reverse transcribed into cDNA using the SuperScript III First-Strand Synthesis System (Invitrogen) according to the manufacturer’s instructions. mRNA expression was measured by SYBR-Green quantitative real-time RT-PCR using the Rotor-Gene Q thermal cycler (Qiagen, Valencia, CA, USA). The primers used for PCR amplification were: CD133 forward, TTTCAAGGACTTGCG AACTCTCTT and reverse, GAACAGGGATGATGTTGGG TCTCA (167 bp); OPN forward, AGACCTGACATCC AGTACCCTG and reverse, GTGGGTTTCAGCACTCTGGT (188 bp). The PCR reaction was carried out in 25 μl buffer, containing 50 ng cDNA, 1 μM of each primer and 12.5 μl 2X Rotor-Gene SYBR-Green PCR Master Mix (Qiagen). The thermal cycling conditions were as follows: denaturation at 95°C for 5 min, followed by 40 cycles of denaturation for 10 sec at 95°C and annealing and extension for 15 sec at 60°C. As the housekeeping gene, glyceraldehyde-3-phosphate dehydrogenase (GADPH; QuantiTect Primer assay, Qiagen) was amplified in order to normalise the amount of total RNA present in each reaction. The quantification of the transcripts was carried out utilizing the dComparative QuantitationT software supplied with Rotor-Gene Q. Endometrial tissue from a normal subject was used as calibrator, and the mean efficiency of the take-off point of the cycling curves was used to calculate the fold change according to the formula: fold change= efficiency^Ct1–Ct2^, where Ct1 and Ct2 are the take-off values of the cycling curves being compared. Each real-time RT-PCR reaction was conducted in duplicate, in order to evaluate data reproducibility. The results are expressed as means ± SEM and the Student’s t-test was used to compare the means of two samples. Significance was accepted at the 5% level.

### Plasma OPN measurement

Peripheral blood samples were collected from patients with endometriosis and control subjects by venous puncture, and immediately centrifuged at 1,500 × g at +4°C for 10 min. Plasma was stored at −80°C until analysis. Plasma OPN levels were measured using the commercially available Quantikine™ Human Osteopontin ELISA kit (R&D Systems, Minneapolis, MN, USA), according to the manufacturer’s instructions. Samples were run in duplicate, and the results are expressed in ng/ml. Data are presented as the means ± SEM. The Student’s t-test was used to compare the means of two samples. Significance was accepted at the 5% level.

### Immunohistochemical analysis

Five-micrometer-thick paraffin-embedded sections were mounted on silanized slides. Following section deparaffinization and rehydration through a graded ethanol series at room temperature, antigen retrieval was performed in Tris-EDTA buffer (pH 9.0, 30 min) and in citrate buffer (pH 6.0, 20 min, 20 min) for OPN and CD133 immunostaining, respectively. As primary antibodies, rabbit polyclonal anti-OPN, diluted 1:1,000 (AB1870; Chemicon, Temecula, CA, USA) and anti-prominin-1, diluted 1:200 (PAB12663; Abnova, Taipei, Taiwan) were used. All the immunohistochemical steps were carried out by the fully automated Menarini Bond™ autostainer (Menarini Diagnostics, Florence, Italy). For the controls, the primary antibody was substituted with non-immune serum and the primary antibody was omitted, thus incubating the slides only with buffer. For the evaluation of immunoreactivity, staining intensities were scored on the basis of the percentage of positive cells for OPN and CD133 as follows: −, <5%; +, 5–50%; and ++, >50%. Immunohistochemical semiquantitative analysis was performed by comparing the results using the χ^2^ test. A p-value <0.05 was considered to indicate a statistically significant difference.

## Results

### Clinical and demographic features of patients with endometriosis and control subjects

Table I displays the demographic and clinical characteristics of the patients and the control subjects. The mean age of the patients affected by endometriosis and the controls was 36.45 years (SD ±9.18) and 33.77 years (SD ±8.09), respectively. The mean number of pregnancies, as well as the parity was not statistically significant between the patients and the healthy control subjects. The difference in BMI between the patients and control groups was not significant. Finally, serum CA125 levels were higher in the patients in comparison to the controls: 59.44±45.56 vs. 19.37±21.97 IU/ml, respectively (p=0.0001).

### mRNA expression of OPN and CD133

**Figure 1:**
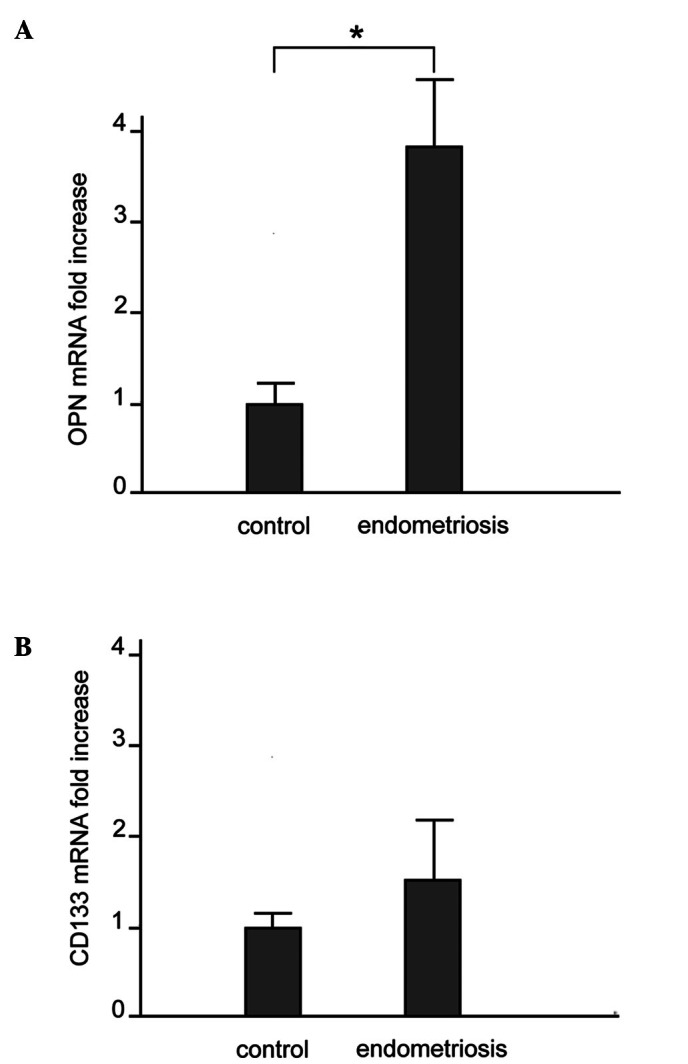
Real-time RT-PCR analysis of osteopontin (OPN) and CD133 mRNA expression in the ectopic (endometriotic) and eutopic (control) endometrium. (A) OPN mRNA expression was significantly higher in patients with endometriosis in comparison to the control subjects. (B) Although CD133 expression was slightly higher in the patients with endometriosis in comparison to the control subjects, the difference was not statistically significant (^*^p<0.01).

The mRNA expression of OPN and CD133 in the control and ectopic endometrial tissues was examined by quantitative real-time RT-PCR. As shown in Fig. 1A, OPN mRNA expression was significantly higher in the patients with endometriosis compared to the controls: (3.79±1.30-fold increase vs. control subjects, p<0.010). A comparison of CD133 mRNA expression between the patients with endometriosis and the controls did not reveal any significant difference (Fig. 1B). A comparison of the OPN mRNA levels between the patients with stage I-II disease and those with stage III-IV disease did not reveal any significant difference (p=0.24) (data not shown).

### Plasma OPN levels

**Figure 2:**
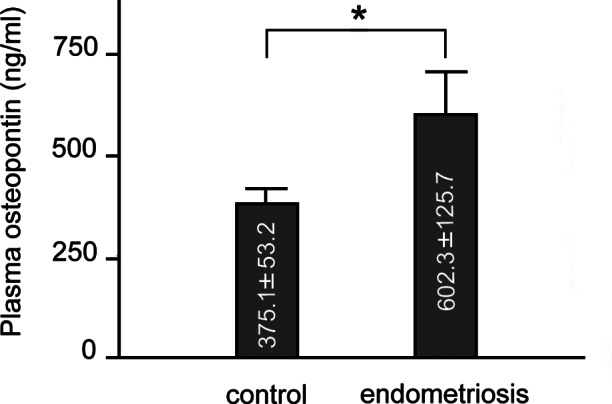
Plasma osteopontin levels in women with endometriosis and control subjects. The mean plasma osteopontin levels were higher in the patients with endometriosis compared to the control group (^*^p<0.01).

As shown in Fig. 2, plasma OPN levels (means ± SEM) were higher in patients with endometriosis in comparison to the controls (602.3±125.7 vs. 375.1±53.2 ng/ml; p<0.01). A comparison of the OPN plasma levels between the patients with stage I-II disease and those with stage III-IV disease and the control group revealed similar results to those obtained between the total number of patients and the control group. Finally, a comparison of the plasma OPN levels between the patients with stage I-II disease and those with stage III-IV disease revealed no significant difference (data not shown). We analysed the correlation between plasma OPN and serum CA125 levels. A positive correlation between plasma OPN and serum CA125 levels was observed in the total number of endometriosis patients (Pearson’s test, r=0.41, p<0.05). However, a comparison between the 2 groups of patients (those with stage I-II and stage III-IV disease) did not reveal any statistically significant difference.

### OPN and CD133 immunohistochemical analysis

**Figure 3:**
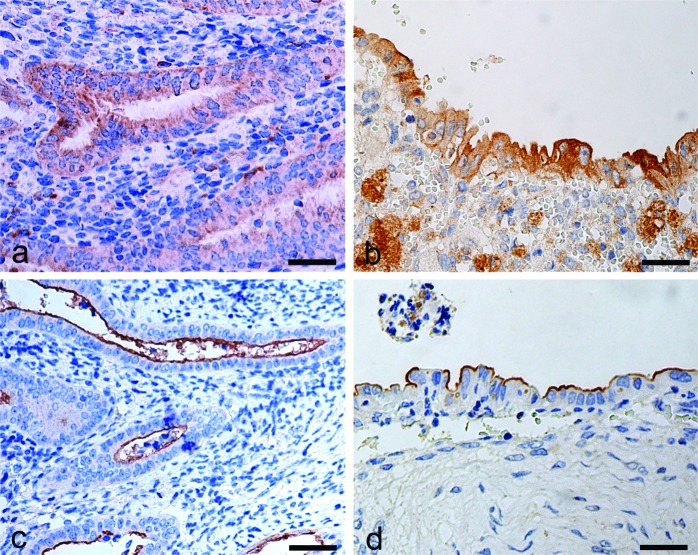
Representative micrographs of immunohistochemistry of osteopontin (OPN) and CD133 expression in the eutopic normal endometrium and endometriotic lesions. (a) In normal tissue, OPN was exclusively immunolocalised in the cytoplasm of epithelium of the functional layer. (b) In endometriotic tissue (stage III-IV), OPN expression was higher in comparison to the control samples, and its localisation was restricted to the cytoplasm of epithelial gland cells, as well as to the macrophages in the stroma. (c) In the control tissue, the surface of epithelial cells lining the lumen was immunostained for CD133. (d) The immunohistochemical pattern for CD133 in endometriotic tissue (stage III-IV) was similar to that obtained in control tissue (bars: a, 80; b, 80; c, 90; and d, 80 μm).

In control sections, OPN immunostaining was localised in the cytoplasm of epithelial cells of the functional layer. Stromal cells were devoid of immunostaining for OPN (Fig. 3a). In the endometriotic tissue, OPN expression was higher in comparison to the control samples, and was localised in the cytoplasm of epithelial gland cells, as well as in several stromal macrophages (Fig. 3b). CD133 immunostaining in the control samples was exclusively localised on the surface of the epithelial cells lining the lumen of the functional layer (Fig. 3c). No immunostaining for CD133 was observed in the stroma. In the endometriotic lesion tissue, the immunohistochemical pattern for CD133 was similar to that observed in the control tissue (Fig. 3d). The results of the analysis of the intensity of the immunohistochemical reactions for OPN and CD133 are summarised in Table II.

### Comparison of clinical findings of patients with endometriosis according to the severity of disease

No significant differences were observed between the age, number of pregnancies, parity, serum CA125 levels and BMI of the patients with endometriosis with stage I-II and stage III-IV disease. In particular, the mean age was 36±2.48 years in the patients with stage I-II disease vs. 35.75±5.94 years in the patients with stage III-IV disease (p=0.96); the number of pregnancies was 1.40±0.40 in the patients with stage I-II disease vs. 1.67±0.89 in the patients with stage III-IV disease (p=0.75); parity was 1.20±0.28 in the patients with stage I-II disease vs. 1.34±0.67 in the patients with stage III-IV disease (p=0.82); serum CA125 levels were 47.71±7.72 IU/ml in the patients with stage I-II disease vs. 72.96±38.74 IU/ml in the patients with stage III-IV disease (p=0.36); BMI was 25.28±3.08 m^2^/kg in the patients with stage I-II disease vs. 20.07±3.30 m^2^/kg in the patients with stage III-IV disease (p=0.33).

## Discussion

The pathological processes involved in endometriosis have not yet been fully elucidated. However, endometriosis has been found to be associated with changes in the expression of several genes, including cytokines (19), such as the multifunctional cytokine OPN, which has been intensively investigated in endometriosis (7,8,10,20).

The results from the present study revealed that OPN mRNA expression, as well as its release in the blood, was significantly increased in endometriotic lesions in comparison to normal tissue. Our findings on OPN mRNA expression are in agreement with those of other studies, which have demonstrated an increased OPN mRNA expression, as well as increased OPN plasma levels in patients with endometriosis in comparison to control subjects, regardless of the phase of the menstrual cycle and diseases stage (8). Similarly, by means of oligonucleotide microarray analysis, complimentary DNA microarrays and quantitative real-time RT-PCR, OPN gene expression has been shown to be increased in endometriotic lesions in a rat model of endometriosis in comparison to normal rats (21,22). On the contrary, with the use of microarray analysis, a downregulation of OPN expression has been observed during the secretory phase in endometriotic lesions (9).

As regards immunohistochemical analysis, we found a higher semiquantitative expression of OPN in both groups of endometriosis patients (the first including patients with stage I-II disease and the second group including patients with stage III-IV disease) in comparison to the eutopic endometrium of the control subjects. Moreover, the OPN staining pattern obtained in the present study, was similar to that observed in the study by Odagiri *et al*(7), who demonstrated that this molecule was mainly immunolocalised in normal and ectopic endometrial epithelial cells. However, these authors did not find a significant difference in the staining intensity between endometriotic lesions and control samples. Moreover, another immunohistochemical study demonstrated different OPN expression intensities in endometriotic lesions in comparison to normal specimens, according to the histological grade (10). Recently, Casals *et al*(20) demonstrated no statistically significant difference in OPN expression between patients and the control subjects.

Such discrepancies may arise from the heterogeneity of the endometriotic samples included in the above studies. For this reason, we restricted the sampling of specimens from women who were in the proliferative phase, and subdivided the patients into 2 groups, one group including those patients with minimal-to-mild disease and the other group including patients with moderate-to-severe disease.

Since it is well known that the functional role of OPN includes cell adhesion, migration, differentiation and regulation of the metastatic spread of tumour cells, it can be hypothesised that the expression of OPN is increased in patients with endometriosis, which would enhance endometrial invasiveness, proliferation and survival in ectopic lesions.

A recent study revealed that a subset of endometriotic cells displayed certain features characteristic of somatic stem cells, such as the presence of CD133 (23). This cell subset seems to originate from bone marrow, and to display endothelial progenitor cell-like features (24). The proliferation and migration of endothelial progenitor cells are influenced by OPN (16,17), which may be required for the homing of these cells (25). OPN shows opposite effects depending on tissue and the physiological state of the cell. In general, OPN is a potent stimulator of cell proliferation, although it represents a negative regulator of hematopoietic stem cell proliferation (26). Possibly, such a mechanism may be mediated by β1-integrins (27), and OPN may induce a lateral organization of lipids and membrane proteins in lipid rafts (28), in order to activate associated signal transduction pathways (29). Although the role of CD133 in stem cells remains unclear, there is much evidence suggesting that this protein plays a potential role as an organiser of specific membrane domains (13), which seems essential for the maintenance of stem cell properties (30). Although we observed the presence of CD133^+^ cells in the normal endometrium, as well as in endometriosis specimens, we did not observe a significant quantitative variation of this protein in patients with endometriosis. However, the presence of the somatic stem cell marker, CD133, suggests the occurrence of a stem cell origin in the pathogenesis of the disease. There is emerging evidence for the occurrence of endometrial cancer stem cells within CD133^+^ and side population cells (31), which could explain the fact that endometriosis is associated with 10–15% of ovarian cancer cases (32).

Thus, further studies are required to determine whether CD133 is expressed and localised in endometrial stem cells, and whether this molecule may be used as a marker of stemness in the eutopic and ectopic endometrium.

In conclusion, the results from our study confirm that OPN is involved in the development of endometriosis by enhancing the invasiveness, proliferation and survival of endometrial cells in ectopic lesions. Furthermore, we suggest that the protein, CD133, cannot be used as a disease marker for endometriosis. Future studies are required in order to further clarify the possible role and specific mechanisms of action of these molecules in the pathogenesis of endometriosis, in order to improve the quality of life of patients with this debilitating and complex disease.

## Figures and Tables

**Table I:** Demographic and clinical characteristics of the patients with endometriosis and the control subjects.

Characteristics	Control subjects (n=30)	Patients with endometriosis (n=31)	P-value
Age, years	33.77±8.09	36.45±9.18	0.25
Number of pregnancies	0.74±1.02	0.93±1.39	0.56
Parity	0.59±0.89	0.86±1.27	0.36
Serum CA125 levels (IU/ml)	19.37±21.97	59.44±45.56	0.0001
BMI m^2^/kg	24.51±2.53	23.32±7.56	0.44

Values are expressed as the means ± SD. The Student’s t-test was used for statistical comparisons between 2 groups. BMI, body mass index.

**Table II:** Evaluation of immunostaining intensity of OPN and CD133 in normal tissue and endometriotic lesions.

		Staining intensity							
		
		OPN				CD133			
			
	n	−	+	++		−	+	++	
Control endometrium	30	3	27	0	-	5	24	1	-
Stage I-II endometriosis	22	13	9	0	p<0.01^a^	4	18	0	NS
Stage III-IV endometriosis	9	0	6	3	p<0.01^a^	2	7	0	NS

Three ranges are utilised: −, <5%; +, 5–50%; and ++, >50%. A comparison of the results was performed using the χ^2^ test (^a^statistically significant difference; NS, not significant). OPN, osteopontin; n, number of patients.
